# Supplementary material for: Clinical Characterization and Predictive Factors for Progression in a Cohort of Patients with Interstitial Lung Disease and Features of Autoimmunity: The Need for a Revision of IPAF Classification Criteria
Source: Medicina (Kaunas). 2023 Apr 19;59(4):794. doi: 10.3390/medicina59040794 (PMC10146211; doi:10.3390/medicina59040794)
Supplement: Supplementary file 1 [file medicina-59-00794-s001.zip › medicina-2223329-supplementary.pdf]

Table S1: Classification of 37 IPAF patients.

CLINICAL DOMAIN	16 (43.2)
Mechanic's hand	1 (2.7)
Distal digital tip ulceration	0 (0)
Inflammatory arthritis or polyarticular morning joint stiffness ≥60 min	9 (24.3)
Palmar teleangiectasia	5 (13.5)
Raynaud phenomenon	6 (16.2)
Unexplained digital oedema	1 (2.7)
Gottron's sign	0 (0)
SEROLOGICAL DOMAIN	31 (83.8)
ANA ≥ 1:320 or any titre for centromeric or nuclear pattern	16 (43.2)
RF ≥ 2 times above the upper limit	4 (10.8)
Anti-CCP	3 (8.1)
Anti-dsDNA	0 (0)
Anti-Ro (SS-A)	10 (27.0)
Anti-La (SS-B)	0 (0)
Anti-ribonucleoprotein (anti-RNP)	0 (0)
Anti-Smith (anti-Sm)	0 (0)
Anti-topoisomerase (Scl 70)	0 (0)
Anti-tRNA synthetase	4 (10.8)
Anti-Pm/Scl	6 (16.2)
Anti-MDA-5	0 (0)
MORPHOLOGICAL DOMAIN	33 (89.2)
NSIP	21 (56.8)
OP	4 (10.8)
NSIP + OP	1 (2.7)
LIP	0 (0)
ILD + multicompartment involvement	7 (18.9)
COMBINATION OF DOMAINS	
Clinical and morphological domain	5 (13.5)
Serological and morphological domain	18 (48.6)
Clinical and serological domain	4 (10.8)
All three domains	10 (27.0)
OTHER MANIFESTATIONS	
Sicca syndrome (confirmed by tear-and salivary tests)	3 (8.1)
Anti-phospholipid antibodies (at high-titre in 2 measurements)	6 (16.2)
MSA/MAA antibodies (e.g. anti-SRP, anti-Mi2a e Mi2b)	8 (21.6)
ANCA (anti-MPO)	1 (2.7)
Anti-Ro52	8 (21.6)

Data are presented as number (%).
Legend: ANA, antinuclear antibody; RF, rheumatoid factor; anti CCP, anti-cyclic citrullinated peptide antibody; anti-MDA-5, anti-melanoma differentiation-associated gene 5; NSIP, nonspecific interstitial pneumonia; OP, organizing pneumonia; LIP, lymphocytic interstitial pneumonia; ILD, interstitial lung disease; MSA, myositis specific antibodies; MAA, myositis associated antibodies; ANCA, anti-neutrophil cytoplasmic antibodies; anti-myeloperoxidase antibodies . See the section “Material and Methods” for further information.

Table S2: Prevalence of autoantibody positivity among IPAF, CTD-ILD and UIPAF patients.

	IPAF	CTD-ILD	UIPAF	*p*-value
ANA ≥ 1:320 or any titre for centromeric or nuclear pattern	16 (43.2)	19 (35.8)	6 (54.5)	0.79
RF ≥ 2 times above the upper limit	4 (10.8)	8 (15.1)	1 (9.1)	0.91
Anti-CCP	3 (8.1)	4 (7.5)	0 (0)	1
Anti-dsDNA	0 (0)	2 (3.8)	0 (0)	0.61
Anti-Ro (SS-A)	10 (27.0)	11 (20.8)	0 (0)	0.17
Anti-La (SS-B)	0 (0)	2 (3.8)	0 (0)	0.61
Anti-Ro52	8 (21.6)	5 (9.4)	0 (0)	0.10
Anti-ribonucleoprotein (anti-RNP)	0 (0)	1 (1.9)	0 (0)	1
Anti-Smith (anti-Sm)	0 (0)	0 (0)	0 (0)	1
Anti-topoisomerase (Scl-70)	0 (0)	1 (1.9)	0 (0)	1
Anti-tRNA synthetase	4 (10.8)	7 (13.2)	1 (9.1)	1
Anti-Pm/Scl	6 (16.2)	2 (3.8)	0 (0)	0.09
Anti-MDA-5	0 (0)	0 (0)	0 (0)	1

Data are presented as number (%).
Legend: ANA, antinuclear antibody; RF, rheumatoid factor; anti CCP, anti Cyclic Citrullinated Peptide antibody; anti-MD-5, anti-melanoma differentiation-associated gene 5.

Table S3: Clinical characteristics of IPAF patients progressed to CTD-ILD or UIPAF during the follow-up.

Patient	Clinical characteristics	New feature	New diagnosis
P1	Female, 67 years, ever smoker, NSIP pattern, anti Jo-1 positivity	Arthritis	Antisynthetase Syndrome
P2	Female, 64 years, former smoker, NSIP pattern, hand arthritis	Sicca syndrome with positive MSGB	Sjogren Syndrome
P3	Female, 65 years, former smoker, fibrotic NSIP pattern, RF ≥ 2 times above the upper limit	UIP	UIPAF
P4	Male, 72 years, former smoker, OP pattern, RF ≥ 2 times above the upper limit, anti CCP positivity	Arthritis	Rheumatoid arthritis
P5	Male, 72 years, former smoker, UIP pattern, ANA 1:640 with granular pattern, anti-Ro52, hand oligoarthritis	Sicca syndrome with positive MSGB	Sjogren Syndrome
P6	Male, 61 years former smoker, NSIP pattern, ANA 1:80 with nucleolar pattern	UIP	UIPAF

As positive MSGB, we considered labial salivary gland with focal lymphocytic sialadenitis and focus score of ≥1 foci/4 mm2. See "Materials and methods" for further information.
Legend: NSIP, nonspecific interstitial pneumonia; MSGB, minor salivary gland biopsy; RF, rheumatoid factor; UIP, usual interstitial pneumonia; UIPAF, "usual" interstitial pneumonia with autoimmune features; OP, organizing pneumonia; anti-CCP, anti-Cyclic Citrullinated Peptide antibody; ANA, antinuclear antibody.
